# Primary vs. Secondary Heart Failure Diagnosis: Differences in Clinical Outcomes, Healthcare Resource Utilization and Cost

**DOI:** 10.3389/fcvm.2022.818525

**Published:** 2022-03-17

**Authors:** Héctor Bueno, Clara Goñi, Rafael Salguero-Bodes, Beatriz Palacios, Lourdes Vicent, Guillermo Moreno, Nicolás Rosillo, Luis Varela, Margarita Capel, Juan Delgado, Fernando Arribas, Manuel del Oro, Carmen Ortega, Jose L. Bernal

**Affiliations:** ^1^Servicio de Cardiología, Hospital Universitario 12 de Octubre, Madrid, Spain; ^2^Spanish National Centre for Cardiovascular Research, Madrid, Spain; ^3^Facultad de Medicina, Universidad Complutense de Madrid, Madrid, Spain; ^4^Centro de Investigación Biomédica en Red en Enfermedades Cardiovasculares (CIBERCV), Madrid, Spain; ^5^Department of Management Control, Hospital Universitario 12 de Octubre, Madrid, Spain; ^6^AstraZeneca, Madrid, Spain; ^7^Instituto de Salud Carlos III (ISCIII), Madrid, Spain; ^8^Facultad de Enfermería, Fisioterapia y Podología, Universidad Complutense de Madrid, Madrid, Spain; ^9^Department of Preventive Medicine, Hospital Universitario 12 de Octubre, Madrid, Spain

**Keywords:** heart failiure, secondary diagnosis, outcome, mortality, resource utilization, cost

## Abstract

**Background:**

There is scarce information on patients with secondary heart failure diagnosis (sHF). We aimed to compare the characteristics, burden, and outcomes of sHF with those with primary HF diagnosis (pHF).

**Methods:**

Retrospective, observational study on patients ≥18 years with emergency department (ED) visits during 2018 with pHF and sHF in ED or hospital (ICD-10-CM) diagnostic codes. Baseline characteristics, 30-day and 1-year mortality, readmission and re-ED visit rates, and costs were compared between sHF and pHF.

**Results:**

Out of the 797 patients discharged home from the ED, 45.5% had sHF, and these presented lower 1-year hospitalization, re-ED visit rates, and costs. In contrast, out of the 2,286 hospitalized patients, 55% had sHF and 45% pHF. Hospitalized sHF patients had significantly (*p* < 0.01) greater comorbidity, lower use of recommended HF therapies, longer length of stay (10.8 ± 10.1 vs. 9.7 ± 7.9 days), and higher in-hospital and 1-year mortality (32 vs. 25.8%) with no significant differences in readmission rates and lower 1-year re-ED visit rate. Hospitalized sHF patients had higher total costs (€12,262,422 vs. €9,144,952, *p* < 0.001), mean cost per patient-year (€9,755 ± 13,395 vs. €8,887 ± 12,059), and average daily cost per patient.

**Conclusion:**

Hospitalized sHF patients have a worse initial prognosis, greater use of healthcare resources, and higher costs.

## Introduction

Although hospitalizations of patients with heart failure (HF) as the main cause of admission are very frequent, the number of patients who need hospitalization for reasons different from HF but who have HF as a secondary diagnosis is actually higher, accounting for a large proportion of all the patients with HF who are hospitalized (1–4). While hospitalizations with a primary diagnosis or any diagnosis of HF (primary or secondary) have been extensively studied, the information regarding hospitalizations with a secondary diagnosis of HF is scarce (1–3). The study of these patients is important as they are, likely, different and, therefore, differences in clinical outcomes, use of healthcare resources, and costs between them can be expected. This study aims to characterize the burden of secondary HF (sHF), comparing the clinical characteristics, outcomes, utilization of healthcare resources, and economical costs, of these patients with those presenting with primary HF (pHF).

## Materials and Methods

This is an observational and retrospective cohort study.

### Patients

All patients aged 18 years or older who had at least one visit to the adult emergency department (ED) of Hospital Universitario 12 de Octubre, Madrid, Spain, during the calendar year 2018 (January 1–December 31, 2018) were selected, and all patients with HF in any position in the diagnosis code either during the ED stay or hospitalization (there, only when HF was codified as present on admission) were included. New onset HF diagnoses in hospitalized patients, patients with K77 code in the ED database in whom the position of the diagnosis, primary or secondary, could not be defined, and patients with unavailable or inconsistent information were excluded.

### Heart Failure Diagnosis

Primary and secondary diagnoses were obtained either from Blecker et al. (1) the ED database (International Classification of Primary Care, 2nd edition, K77 code) or (2) the Admission and Emergency Minimum Basic Datasets (Inclusion criterion: International Classification of Diseases, Tenth Revision, Clinical Modification codes I11.0*, I13.0*, I13.2*, and I50*). Patients with a primary diagnosis of HF formed the pHF group, and those with one other different primary diagnosis but having HF as a non-primary diagnosis formed the sHF group.

### Outcomes and Healthcare Resource Use

Information on clinical outcomes and use of healthcare resources was obtained by analysis of the hospital’s patient management information system, which integrates electronic health records and most of the administrative data. Mortality and healthcare resource utilization after discharge were registered during the following year after the ED or hospital discharge through the online Madrid regional population information system (CIBELES) that gives information on vital status and recording (all-cause) mortality at a population level. This database is part of the Madrid regional healthcare service (*Servicio Regional de Salud de Madrid, SERMAS*) information system.

Emergency department visits were considered as any medical attention in the hospital ED regardless of the time spent and the final disposition. Any visit to the hospital ED for any reason after the index ED visit discharge was computed as a re-ED visit. The length of ED stay was calculated as the date of ED discharge or hospital admission minus ED admission date. Hospital stays were computed as any time in the hospital lasting beyond midnight. Length of hospital stay was calculated as the date of discharge minus the admission date in days. Readmissions were considered as any hospital stay for any reason after discharge from the index hospitalization. Specific cuts were done at 30 and 365 days after index discharge. Outpatient visits were defined as any scheduled medical or nursing consultation; any day-hospital visit or any cardiac rehabilitation visit (classified each as first or subsequent).

The Clinical Outcomes, HEalthcare REsource utilizatioN, and relaTed costs (COHERENT) model were used for the representation of the mortality-healthcare resource utilization composite outcome and for cost calculation (5). In brief, this new system to evaluate complex composite outcomes by graphical models is constructed developing a hierarchical code system with a mutually exclusive list of potentially relevant clinical situations defined as the patient clinical status (alive or dead) and location (i.e., at home, ED, or in hospital), which are computed daily during each defined period.

The trajectory of the cohort is represented in an area graph plotting the percentage of patients in each possible clinical situation represented in a set of stacked colored vertical columns on the *Y*-axis, each column representing 100% of observed patients and each color representing the percentage of patients in each clinical situation, with each day of follow-up on the *X*-axis. Time points were assumed to be full days. The number of clinical situation categories shown in the graph can be personalized, from a basic model (at home, in hospital, and dead) to a comprehensive model showing all departments involved. The graph was designed with R Project for Statistical Computing, version 4.0.3 (2017 The R Foundation for Statistical Computing, Vienna, Austria) (6).

### Cost Calculation

Data provided by the Accountability Department of Hospital Universitario 12 de Octubre were used for reporting costs for clinical situations (i.e., in hospital, in the ED, and in day-hospital). This method consists of a full cost system in which the cost of each episode is calculated by the addition of all costs imputable to the patient or the episode (housing, diets, drugs, and devices), the unitary costs of each product or activity included in the hospital service catalog (laboratory analysis, diagnostic and therapeutic interventions, operating room times, postoperative recovery unit stays) and all other costs that cannot be directly imputed to the patient or the episode (residual cost), which are transferred to the clinical episode cost through the indirect imputation criteria. Hospital Universitario 12 de Octubre belongs to the Spanish Network of Hospital Costs (RECH is the original Spanish term), an initiative for the dissemination and the study of the set of costs related to hospital activities at the patient level, which was the foundation for calculating the weights and costs of healthcare processes for patients attended in the network of hospitals belonging to the National Health System (7). Thanks to this method, daily estimated and cumulative costs were calculated for episodes of care and for the patient journey, which in this case includes all admission, emergency, and day-hospital episodes that a patient with a medical problem experiences over the observation time (365 days here) of the patients of both cohorts. Other costs, such as total cost distribution and mean cost per episode, were calculated as well. Thus, the burden of each clinical situation in the overall cost is perfectly reflected.

### Statistical Analysis

Categorical variables are presented as number (%) and continuous variables and as mean ± *SD* or median (interquartile range) for normally and non-normally distributed, respectively. The participants’ characteristics were compared by Student’s *t*-test, the *U* Mann–Whitney test, analysis of variance, or the Kruskal–Wallis test, when appropriate for continuous variables and by the chi-square test for categorical variables. Survival curves are estimated using the Kaplan–Meier method and compared statistically using the log-rank test. Costs are presented as absolute expenditures (in euros) and percentage of total cost by follow-up time, units of patient care, and mean per patient and day. For all tests, values of *p* < 0.05 were considered statistically significant.

### Ethics

The study complies with the Declaration of Helsinki and was approved by the Hospital Universitario 12 de Octubre Ethics Committee.

## Results

### Patients and Global Outcomes

Between January 1 and December 31, 2018, there were 192,733 ED visits from 123,187 patients, of which 93,962 (48.8%) visits from 66,551 patients were for medical reasons. A primary or secondary diagnosis of HF was registered in 2,557 (3.8%) patients in the ED. In addition, 723 (1.1%) patients who did not have an HF diagnosis in the ED diagnostic code but were admitted to the hospital and at discharge had an HF diagnosis present on admission were included. In total, 3,280 (4.9%) patients who visited the ED admitted in the hospital had one HF diagnosis. Of the 2,557 patients, 182 (7.1%) patients with an HF diagnosis but in whom it could not be established whether it was a primary or a secondary diagnosis were excluded from this study. From 2,375 valid patients with an HF diagnosis in the ED, 1,563 (65.8%) were hospitalized, 797 (33.6%) were discharged home directly from the ED, and 15 (0.6%) died in the ED (Supplementary Figure 1).

Among the 797 patients discharged home after the index ED visit, mortality rates were 2.5% (*n* = 20) at 30 days and 18.3% (*n* = 146) at 1 year. First hospitalization rates were 10.4% (*n* = 83) at 30 days and 46.2% (*n* = 369) at 1 year, and recurrent ED visits 22.8% (*n* = 182) at 30 days and 70.0% (*n* = 558) at 1 year.

There were 2,286 eligible hospitalized patients, 1,563 with an HF diagnosis in the ED, and 723 with an HF diagnosis as present on admission but not coded in the ED (Supplementary Figure 1). Of these, 203 (8.8%) died in hospital, 222 (9.7%) at 30 days, and 669 (29.3%) at 1 year. The median length of stay was 8 days (6–12). Readmission rates were 13.4% (*n* = 306) and 45.3% (*n* = 1036) at 30 days and 365 days, respectively. Rates of 30-day and 1-year re-ED visit were 16.3% (*n* = 374) and 58.6% (*n* = 1341), respectively.

### Primary vs. Secondary Heart Failure in Patients Discharged Home From the Emergency Department

Of the 797 patients discharged home from the ED, 434 patients (54.5%) presented pHF and 363 (45.5%) sHF. There were no major differences in several baseline characteristics but a greater proportion of patients with sHF presented with comorbidities, atrial fibrillation, chronic obstructive pulmonary disease, and respiratory failure in particular (Supplementary Table 1). The main diagnoses for patients discharged from the ED with sHF are shown in Supplementary Table 2. These were most often heart rhythm disturbances, acute respiratory disease, and infections. The mean length of index ED stay was 1.66 ± 0.67 days in patients with sHF *vs*. 1.66 ± 0.69 days in patients with pHF (*p* = 0.882). The 30-day and 1-year mortality rates were 1.8 and 16.6%, respectively, for patients with pHF, and 3.3 and 20.4%, respectively, for patients with sHF (*p* = 0.27 and 0.20, respectively, for the comparisons between pHF and sHF). There were no significant differences between patients with pHF and sHF in 30-day hospitalization rates (11.8 *vs*. 8.8%, *p* = 0.22) and 30-day new ED visits (24.2 *vs*. 21.2%, *p* = 0.36) but patients with pHF presented higher 1-year rates of hospitalization (50.9 *vs*. 40.7%, *p* = 0.005) and new ED visits (73.9 *vs*. 65.2%, *p* < 0.009). The median number of days spent at home after discharge was lower for patients with pHF (345 [314–356 *vs*. 346 [308–357, *p* = 0.96]). Patients with sHF had significantly lower (*p* < 0.001) economical costs: total cost (€2,247,217 *vs*. €1,416,579), mean cost per patient journey (€3,902.4 ± 6,997.5 *vs*. €5,177.9 ± 9,537.3), and mean cost per patient per day (€10.69 ± 126.49 *vs*. €14.18 ± 151.82) (Supplementary Table 3).

### Primary vs. Secondary Heart Failure in Hospitalized Patients

**FIGURE 1:**
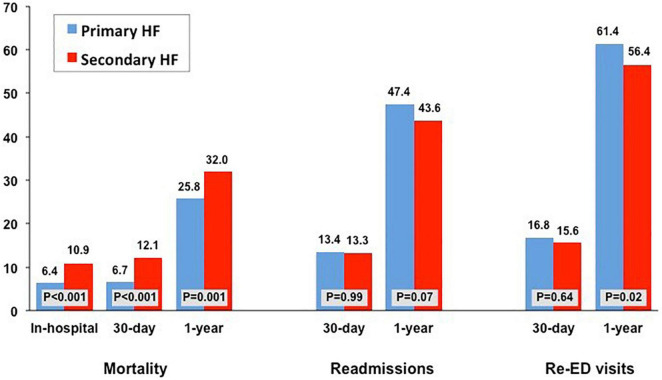
Rates of in-hospital, 30-day and 1-year mortality rates, 30-day and 1-year readmission rates, and 30-day and 1-year new emergency department (ED) visits according to the type of heart failure diagnosis: primary and secondary.

Among the 2,286 patients hospitalized, 1,029 (45.0%) were pHF and 1,257 (55.0%) sHF. Of the 723 patients who did not have an HF diagnosis in the ED but had a final diagnosis of HF as present on admission, 134 were pHF and 589 were sHF. Baseline characteristics of hospitalized patients are shown in Table 1. Compared with pHF hospitalizations, hospitalized patients with sHF had a lower predominance of women and greater non-cardiovascular comorbidity, including cancer, respiratory, and renal disease, with no differences in age and risk factors. HF with reduced ejection fraction was more frequent in pHF while the unavailability of left ventricular ejection fraction was more frequent among patients with sHF. The most frequent main diagnoses for hospitalized patients with sHF are shown in Supplementary Table 4. These diagnoses were for the most often infections and acute respiratory diseases. Patients with sHF were admitted less often in the cardiology department (131 [10.4%] *vs*. 229 [22.2%]; *p* < 0.001) and more often in any other department: internal medicine (1,054 [83.8%] *vs*. 779 [75.7%], *p* < 0.001), general or cardiac intensive care unit (35 [2.8%] *vs*. 8 [0.7%], *p* < 0.001), and other departments (37 [2.9%] *vs*. 13 [1.2%]; *p* < 0.001). The mean length of index hospital stay was longer for hospitalizations with sHF than for pHF (10.8 ± 10.1 *vs*. 9.7 ± 7.9 days, *p* = 0.001). The proportion of patients with sHF who were treated with angiotensin inhibitors, beta-blockers, and mineralocorticoid-receptor antagonists during hospitalization was lower (Table 1). The in-hospital mortality was higher in patients with sHF (10.9 *vs*. 6.4%, *p* < 0.001; Figure 1).

**TABLE 1:** Baseline characteristics in hospitalized patients.

	Primary HF	Secondary HF	

	(*n* = 1029)	(*n* = 1257)	*P*-value
Age, years (mean ± SD)	81.11 ± 11	81.25 ± 11	0.528
Female sex, n (%)	607 (58.9%)	670 (53.3%)	0.007
SBP, mmHg (mean ± SD)	134.4 ± 20	129.5 ± 21	<0.001
DBP, mmHg (mean ± SD)	70.4 ± 14	66.6 ± 13	<0.001
HR, bpm (mean ± SD)	80.7 ± 19	82.5 ± 19	0.023
**Risk factors, n (%)**			
Hypertension	804 (78.1%)	967 (76.9%)	0.970
Dyslipidaemia	430 (41.7%)	535 (42.5%)	0.741
Diabetes	455 (44.2%)	556 (44.2%)	0.990
Smoking	323 (31.4%)	440 (35%)	0.075
**Comorbidities, n (%)**			
Ischemic heart disease	172 (16.7%)	203 (16.1%)	0.759
Hypertensive heart disease	102 (10%)	275 (21.8%)	<0.001
Chronic kidney disease	78 (7.5%)	192 (15.2%)	<0.001
Atrial fibrillation	236 (22.9%)	345 (27.4%)	0.015
Heart valve disease	71 (6.8%)	111 (8.8%)	0.105
COPD	48 (5%)	185 (14.7%)	<0.001
Cancer	20 (1.9%)	80 (6.3%)	<0.001
Respiratory failure	414 (40.2%)	610 (48.5%)	<0.001
Charlson index, (mean ± SD)	1.18 ± 0.49	1.6 ± 0.81	<0.001
Charlson index > 2, n (%)	26 (2.5%)	160 (12.7%)	<0.001
**Left ventricular ejection fraction, n (%)**			<0.001
<40%	165 (16%)	111 (8.8%)	
40-50%	109 (10.6%)	95 (7.5%)	
>50%	676 (65.6%)	864 (68.7%)	
Information not available	79 (7.7%)	156 (12.4%)	
**In-hospital medical therapies**			
Beta-blockers	655 (63.65%)	596 (47.41%)	<0.001
RAAS inhibitors			
ACE inhibitors	472 (45.87%)	435 (34.61%)	<0.001
ARB	229 (22.25%)	218 (17.34%)	0.004
ARNI	27 (2.62%)	11 (0.88%)	0.002
MRA	335 (32.56%)	239 (19.01%)	<0.001
Diuretics	984 (95.63%)	1121 (89.18%)	<0.001
Inotropic agents	21 (2.04%)	35 (2.78%)	0.302
**Antithrombotic drugs**			
Aspirin	297 (28.86%)	398 (31.66%)	0.132
P2Y12 inhibitors	111 (10.79%)	164 (13.05%)	0.10
Oral anticoagulants	585 (56.85%)	516 (41.05%)	<0.001
Lipid-lowering drugs	557 (54.13%)	615 (48.93%)	0.019
**Antidiabetic therapies**			
Insulin	448 (43.54%)	551 (43.83%)	0.818
Metformin	40 (3.89%)	43 (3.42%)	0.651
Other antidiabetic drugs	52 (5.05%)	29 (2.31%)	<0.001

*RAAS, Renin-angiotensin-aldosterone system; ACE, angiotensin-converting enzyme; ARB, angiotensin receptor blocker; ARNI, angiotensin receptor neprilysin inhibitor MRA, mineralocorticoid receptor antagonist.*

**FIGURE 2:**
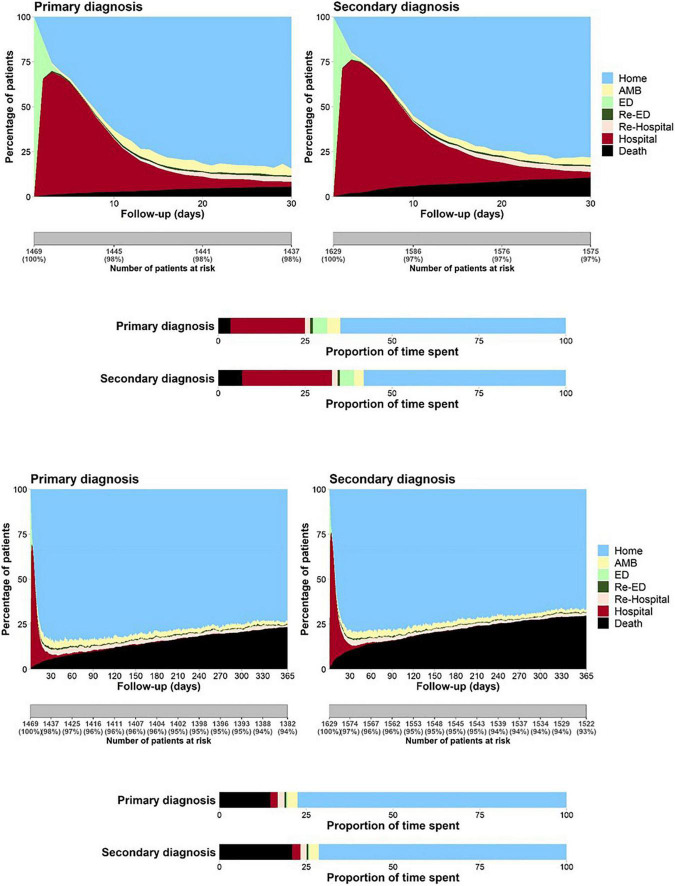
The 30-day (top) and 1-year (down) composite outcomes using the COHERENT model according to the type of heart failure diagnosis: primary (left) and secondary (right). The black area represents mortality. The light green area on the left side of each figure represents the time spent in the first emergency department (ED) visit, the brown area represents the time spent in the first hospitalization, the light blue area in the top right corner represents the time spent at home. The days with one outpatient office visit, the number of days with subsequent ED visits, or the days spent in the hospital during re-hospitalizations are represented by the light yellow, dark green, and light brown areas, respectively. The proportion of time spent in each clinical condition can be compared in the display in the horizontal bars shown below.

**FIGURE 3:**
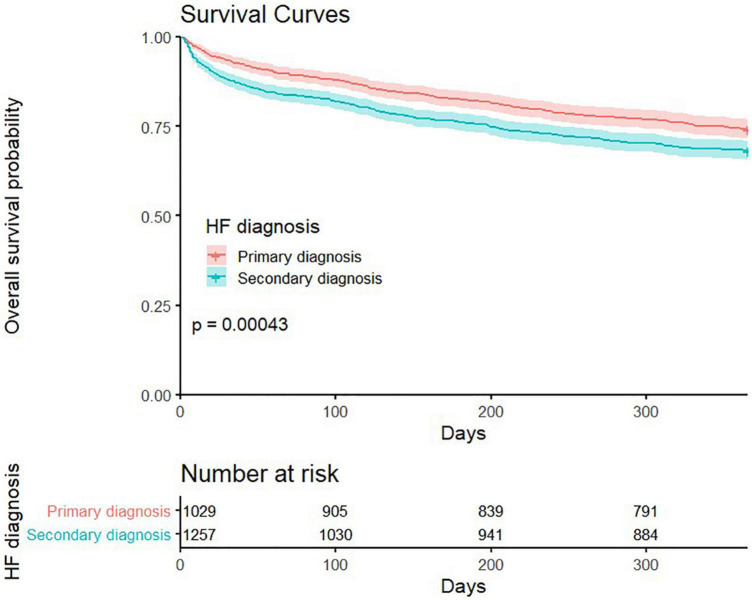
Survival curves for hospitalized patients according to the type of heart failure diagnosis: primary and secondary.

The cumulative time spent in hospital or ED throughout the year was similar in both groups (median days in hospital, 12 [*IQR*: 6–23] for pHF *vs*. 13 [*IQR*: 7–23] for sHF, *p* = 0.65) and in the ED (median days with ED visit, 2 [*IQR*: 1–4] for pHF *vs*. 2 [*IQR*: 1–3] for sHF, *p* = 0.44) (Figure 2). However, the median number of days spent at home was lower for patients with sHF (323 [153–346] *vs*. 329 [257–346] days, *p* < 0.001), accounting for 88.5 and 90.1% of the time alive out of hospital, respectively (*p* = 0.02). The 30-days and 1-year mortality rates were higher in patients with hospitalizations with sHF than in those with pHF (12.1 *vs*. 6.7%, *p* < 0.001 and 32 *vs*. 25.8%, *p* < 0.001; Figures 1, 3), mainly driven by the higher initial mortality. The use of healthcare resources and economical costs are shown in Figure 2 and Table 2. Readmission rates at 30 days were 13.3% (*n* = 168) for hospitalizations with sHF and 13.4% (*n* = 138) for pHF (*p* = 0.99) and at 365 days were 43.6% (*n* = 548) for hospitalizations with sHF and 47.4% (*n* = 488) for pHF (*p* = 0.073). Re-visits to the ED at 30 and 365 days were 15.6% (*n* = 201) for hospitalizations with sHF and 16.8% (*n* = 173) for pHF (*p* = 0.637) and 56.4% (*n* = 709) for sHF and 61.4% (*n* = 632) for pHF (*p* = 0.017), respectively. The total numbers of outpatient visits after the HF hospitalization were 14,396 in sHF patients and 13,004 in pHF patients, with a mean number of outpatient visits throughout the following year of 11.4 ± 11.7 after hospitalizations with sHF and 12.6 ± 11.1 after pHF hospitalizations (*p* < 0.001).

**TABLE 2:** Costs per patient journey, clinical status and episode during the first year in hospitalized patients.

	Primary HF			Secondary HF			
		
	(*n* = 1029)			(*n* = 1257)			
		
	Frequencies	Cost (euros)	%	Frequencies	Cost (euros)	%	*P*-value
Total patient-days, n	375,585		100%	458,805		100%	–
Total cost		9,144,952	100%		12,262,422	100%	–
Mean cost per patient journey		8,887.2 ± 12,059.2			9,755.3 ± 13,395.3		<0.001
Mean cost per patient per day		24.34 ± 33.03			26.73 ± 36.7		<0.001
Use and cost per clinical status							
Mean days spent at ED	2.77 ± 2.32			2.62 ± 2.38			0.436
Emergency Department		882,295.1	9.64%		1,035,105.6	8.44%	–
Mean days spent in-hospital	17.7 ± 18.1			18.0 ± 17.9			0.647
Total costs for hospitalizations		8,030,614.7	87.81%		10,628,470.6	86.67%	–
Index hospitalizations		4,020,987.5	43.96%		5,852,662.5	47.72%	–
Readmissions		4,009,627.2	43.84%		4,775,808.1	38.94%	–
Day-Hospital		232,041.8	2.53%		598,846.2	4.88%	–
Mean cost per episode		1,856.84			2,010,89		0.508
Number of episodes	4,925		100%	6,098		100%	
Emergency Department	2,571	343.2 ± 141.8	52.2%	3,013	343.5 ± 141.7	49.4%	0.755
Day-hospital	427	543.4 ± 944.9	8.67%	812	737.5 ± 1,298.1	13.3%	0.006
Hospital	1,927	4,167.4 ± 6,682.1	39.12%	2,273	4,676.0 ± 6,951.9	37.3%	<0.001
Index hospitalizations	1,029	3,907.7 ± 6,323.2	20.89%	1,257	4,656.1 ± 6,532.3	20.6%	<0.001
Readmissions	898	4,465.1 ± 7,057.2	18.23%	1,016	4,700.6 ± 7,440.7	16.6%	0.310

Contrary to the patients discharged home directly from the ED, the total 1-year healthcare-related cost for all patients was higher for sHF compared with pHF (€12,262,422 *vs*. €9,144,952). Similarly, the mean cost per patient journey during the year (€9,755.30 ± 13,395 *vs*. €8,887 ± 12,059, *p* < 0.001), and the average daily cost per patient (€26.73 ± 36.70 *vs*. €24.34 ± 33.03, *p* < 0.001) were higher in patients with sHF (Table 2).

## Discussion

Our study shows that patients with sHF pose a great burden on the healthcare system, even greater than those with pHF, particularly when these require hospitalization initially. There are more patients with secondary than with primary HF hospitalizations. These patients have greater comorbidity, worse short-term prognosis, use more healthcare resources, and cause higher costs in absolute and relative terms at 1 year.

The prevalence of sHF found in our study is consistent with that reported in Europe, between 57 and 58% (3, 8), but lower than that described in the United States, between 73 and 76% (1, 2, 9, 10). To our knowledge, all published studies in the field are national so no international comparisons vs. pHF on this aspect are available. We speculate that regional differences in the rates of sHF diagnosis may be explained by different cultures in disease coding rather than by true epidemiological differences but this hypothesis needs confirmation.

Patients with hospitalizations with sHF present a greater degree of comorbidity and their clinical profile is different but not substantially different compared with patients with pHF hospitalization. The main reasons for hospitalization in these patients were most often respiratory, either infections or decompensations of prior respiratory disease. Less frequent were acute myocardial infarction, arrhythmias, or stroke. Contrary to what might be expected, these patients are not older (2, 10, 11) and have no major differences in cardiovascular risk factors (10, 11). While HF with reduced ejection fraction was more frequent in patients with pHF, a larger proportion of patients with sHF had unknown left ventricular ejection. Although this may explain, at least in part, the lower use of HF guidelines-recommended therapies in these patients as these are essentially indicated in the former; other reasons, such as not being HF, the main cause of admission may be associated with this difference. In contrast with prior reports (10), our patients with sHF diagnosis after their first ED visit required hospitalization and intensive care admission more often than those with pHF. What was consistent with prior studies is their longer length of stay (10, 11) and their higher in-hospital mortality (10–12). Interestingly, the survival curves show an early divergence and then run roughly in parallel after the first month. Thus, these patients seem to have an acute clinical challenge, probably related to the acute medical cause of hospitalization, causing the early increase in mortality but they do not do far worse after hospital discharge. Actually, their rates of recurrent ED visits or readmission after 30 days are not different (9, 10), and 1-year rates are actually lower than in patients with pHF. Whether this is explained by a different clinical behavior, competing risks, or other reasons remain unknown. The difference in HF-specific therapies would be an unlikely explanation, as the consequences would increase with time. Interestingly, Erez et al. showed, after a follow-up of 10 years, a 12% lower 10-year adjusted mortality risk in patients with sHF hospitalizations compared with pHF hospitalizations (11), for which there is no clear explanation.

The clinical behavior of patients with sHF suggests that special attention should be paid to these patients during the first month, when a worse outcome may be expected. One of the key factors to consider is the risk of undertreatment, as found in our results. Although the reasons for the difference in evidence-based pharmacological therapies between patients with sHF and pHF cannot be assessed given the study design, this finding suggests that there may be a need to create specific programs for patients presenting with or developing HF during hospitalization for other reasons, particularly in units without HF experts, to optimize their medical management and minimize their risk, including multidisciplinary HF teams with HF specialists, nurses or clinical pharmacists or facilitated pathways for direct collaboration with their primary caregivers (i.e., cardiologists, internists, and geriatricians). Afterward, conventional care for HF patients may be sufficient as their prognosis is not worse (if not better), compared with patients with pHF.

Interestingly, we found that while among the patients discharged home from the ED, the mean economic costs (both per journey and per episode) were lower for sHF than for pHF, the opposite was found among hospitalized patients. This is an original finding as to our knowledge; no previous study had analyzed the impact of sHF from the perspective of the disposition following ED. Several studies characterized the economic impact of HF hospitalizations, a few of them addressing the differences between sHF and pHF hospitalizations. Stewart et al. estimated the cost of HF in the UK approximately as 2% of the total cost of the National Health System in the year 2000, with a greater weight of hospitalization with sHF (8). Likewise, in our study, the total cost of hospitalizations with sHF is 34% higher than that of those for pHF. This difference accounts not only for 22.1% of the extra cost explained by the absolute difference in the number of patients with sHF, but also for the higher mean cost of the trajectory of patients with sHF (9.8%), somewhat higher than the average healthcare-related cost per HF found in prior studies in the same environment (13), although with methodological and temporal differences. In absolute terms, the total economical costs calculated for hospitalizations and the use of healthcare in our study were much lower than those published in the United States (10, 14, 15). These differences may be related to a less expensive healthcare system, a greater efficiency of the system, or methodological differences in cost estimation. Considering the mean cost per episode, index hospitalization costs are 19.2% higher for hospitalizations with sHF, readmission costs are 5.3% higher, and day-hospital 35.7% higher, compared with pHF hospitalizations, with no differences in total ED-related costs. These results are in line with those reported by Wang et al. (14) and Ng et al. (10), who found, respectively, a 45 and 40% higher mean hospitalization cost in hospitalizations with sHF. The fact that the care of patients with sHF requires higher health resources in absolute and relative terms is relevant and consistent with the higher comorbidity burden of these patients, in particular with their longer length of hospital stay, although it is possible that other differences in imputed costs, such as for intermediate products or unitary costs, may have played a role. These aspects deserve further research.

## Limitations

This is a single-center study so our findings may not be generalizable, particularly patterns of care that may change by healthcare systems (i.e., rates of discharge home from ED, length of stay, or readmissions). No cause-specific analysis was performed for primary causes of hospitalization or ED visits in sHF cases or for outcomes (mortality readmissions or ED re-visits). Absolute costs cannot be extrapolated to other countries as well. However, the relative differences in mortality and healthcare resource use may parallel those happening elsewhere. Although there are inherent limitations to the use of Minimum Basic Dataset data as in our retrospective study, the use of administrative information has proven to be valid to estimate outcomes in health services, compared with medical records (16, 17).

## Conclusion

Patients with sHF are a distinct and important group of patients with HF, with worse initial prognosis, greater healthcare resource use, and greater economic cost compared with patients with pHF. These patients have specific features and needs, particularly during the first weeks after ED visit or hospitalization. The reasons for their worse prognosis only in the early phase of the episode suggests a key role of the cause for seeking care but the optimal management of these patients during hospitalization and immediately after needs to be defined. Specific research in improving the management and outcomes of patients with sHF is warranted.

## Data Availability Statement

The data analyzed in this study is subject to the following licenses/restrictions: The data belongs to the database of Hospital 12 de Octubre and must be protected to safeguard the identity of the participants. Requests to access these datasets should be directed to HB, hector.bueno@cnic.es.

## Ethics Statement

The studies involving human participants were reviewed and approved by Hospital 12 de Octubre Review Board. Written informed consent for participation was not required for this study in accordance with the national legislation and the institutional requirements.

## Author Contributions

All authors have contributed to the manuscript, fulfill the required criteria for authorship, and have approved the final version for submission to Frontiers in Cardiovascular Medicine.

## Conflict of Interest

HB receives research funding from the Instituto de Salud Carlos III, Spain (PIE16/00021 and PI17/01799), Sociedad Española de Cardiología, Astra-Zeneca, Bayer, PhaseBio and Novartis; has received consulting fees from Astra-Zeneca, Novartis; and speaking fees from Novartis and MEDSCAPE-the heart.og. BP, LVa, and MC are AstraZeneca Spain employees. JB reports grant from AstraZeneca, during the conduct of the study. FA reports personal fees from Daiichi Sankyo, personal fees from Impulse Dynamics, personal fees from Medtronic, personal fees from Boston Scientific, personal fees from Bayer, personal fees from Bristol Myers Squibb, personal fees from Arrhythmia Network Technology SL/BAROSTIM, personal fees from Abbott, outside the submitted work. JD reports personal fees from Novartis, personal fees from Astra Zeneca, personal fees from Boehringer, outside the submitted work. RS-B reports other from Boston Scientific, non-financial support from Medtronic, personal fees and other from Daichii Sankyo, and other from Abbott, outside the submitted work. The remaining authors declare that the research was conducted in the absence of any commercial or financial relationships that could be construed as a potential conflict of interest.

## Publisher’s Note

All claims expressed in this article are solely those of the authors and do not necessarily represent those of their affiliated organizations, or those of the publisher, the editors and the reviewers. Any product that may be evaluated in this article, or claim that may be made by its manufacturer, is not guaranteed or endorsed by the publisher.
