# Supplementary material for: Primary vs. Secondary Heart Failure Diagnosis: Differences in Clinical Outcomes, Healthcare Resource Utilization and Cost
Source: Front Cardiovasc Med. 2022 Mar 17;9:818525. doi: 10.3389/fcvm.2022.818525 (PMC8967997; doi:10.3389/fcvm.2022.818525)
Supplement: Supplementary file 1 [file Data_Sheet_1.docx]

**SUPPLEMENTARY MATERIAL**

**Supplementary Table 1. Baseline characteristics in patients discharged home from the ED**

|  | **Primary HF**  (n= 434) | **Secondary HF**  (n= 363) | **P value** |
| --- | --- | --- | --- |
| **Demographics** |  |  |  |
| Age, years (mean±SD) | 80.7 ± 10.4 | 79.1 ± 12.4 | 0.149 |
| Female sex, n (%) | 244 (56.2%) | 200 (55.1%) | 0.805 |
| SBP, mmHg (mean±SD) | 139.3 ± 21 | 138.4 ± 20.8 | 0.919 |
| DBP, mmHg (mean±SD) | 70.5 ± 13 | 71.04 ± 14.6 | 0.783 |
| HR, bpm (mean±SD) | 77.9 ± 18.6 | 79.1 ± 18.3 | 0.465 |
| **Risk factors, n (%)** |  |  |  |
| Hypertension | 337 (77.6%) | 281 (77.4%) | 0.99 |
| Dyslipidaemia | 200 (46.1%) | 177 (48.7%) | 0.495 |
| Diabetes | 182 (41.9%) | 133 (36.4%) | 0.147 |
| Smoking | 136 (31.3%) | 127 (34.9%) | 0.31 |
| **Comorbidities, n (%)** |  |  |  |
| Ischemic heart disease | 52 (11.9%) | 36 (10%) | 0.416 |
| Hypertensive heart disease | 14 (3.2%) | 19 (5.2%) | 0.215 |
| Chronic kidney disease | 18 (4.1%) | 14 (3.8%) | 0.978 |
| Atrial fibrillation | 49 (11.3%) | 86 (23.4%) | <0.001 |
| Heart valve disease | 18 (4.1%) | 17 (4.7%) | 0.846 |
| Chronic obstructive pulmonary disease | 7 (1.6%) | 19 (5.2%) | 0.007 |
| Cancer | 10 (2.3%) | 17 (4.7%) | 0.098 |
| Respiratory failure | 50 (11.5%) | 63 (17.3%) | 0.024 |
| Charlson index, (mean±SD) | 1.09 ± 0.3 | 1.18 ± 0.43 | <0.001 |
| Charlson index >2, n (%) | 1 (0.2%) | 6 (1.6%) | - |
| **Left ventricular ejection fraction, n (%)** |  |  | 0.015 |
| <40% | 34 (7.8%) | 22 (6%) |  |
| 40-50% | 39 (8.9%) | 12 (3.3%) |  |
| >50% | 299 (68.8%) | 246 (67.7%) |  |
| Information not available | 62 (14.3%) | 15 (4.1%) |  |
| **Medical therapies*** |  |  |  |
| Beta-blockers (BB) | 126 (29%) | 83 (22.9%) | 0.03 |
| RAAS inhibitors |  |  |  |
| ACE inhibitors | 74 (17.1%) | 41 (11.3%) | 0.378 |
| ARB | 39 (8.9%) | 29 (7.9%) | 0.628 |
| ARNI | 9 (2.1%) | 4 (1.1%) | 0.722 |
| MRA | 75 (17.3%) | 28 (7.7%) | 0.003 |
| Diuretics | 183 (42.2%) | 108 (29.8%) | 0.053 |
| Inotropic agents | 4 (1%) | 7 (1.9%) | - |
| Antiplatelets |  |  |  |
| Aspirin | 57 (13.1%) | 46 (12.6%) | 0.229 |
| P2Y12 inhibitors | 18 (4.1%) | 23 (6.3%) | 0.029 |
| Oral anticoagulants | 117 (26.9%) | 51 (14%) | 0.001 |
| Lipid-lowering drugs | 116 (26.7%) | 74 (20.3%) | 0.953 |
| Metformin | 13 (2.9%) | 8 (2.2%) | 0.99 |
| Other oral antidiabetic drugs | 9 (2%) | 8 (2.2%) | - |
| Insulin | 88 (20.2%) | 67 (18.4%) | 0.218 |

* Information only according to that available in the ED online report. These rates are probably underestimated

**Abbreviations:** RAAS, Renin-angiotensin-aldosterone system; ACE, angiotensin-converting enzyme; ARB, angiotensin receptor blocker; ARNI, angiotensin receptor neprilysin inhibitor MRA, mineralocorticoid receptor antagonist

**Supplementary Table 2. Top 20 primary diagnoses among patients with secondary heart failure diagnosis who were discharged home directly from the emergency department**

| **Rank** | **Primary diagnosis** | **Episodes**  **n (%)** |
| --- | --- | --- |
|  | Atrial fibrillation and flutter | 59 (22.60) |
|  | Oedema | 25 (9.58) |
|  | Other respiratory disorders | 19 |
|  | Acute lower respiratory tract infection | 17 |
|  | Sore throat and chest pain | 15 |
|  | Acute bronchitis | 15 |
|  | Respiratory disturbances | 15 |
|  | Other chronic obstructive pulmonary diseases | 14 |
|  | Heartbeat disturbances | 11 |
|  | Respiratory insufficiency | 10 |
|  | Pneumonia, unspecified microorganism | 8 |
|  | Other anaemia | 8 |
|  | Angina pectoris | 7 |
|  | Acute upper respiratory tract infections of multiple or unspecified location | 7 |
|  | Essential hypertension (primary) | 6 |
|  | Syncope and collapse | 6 |
|  | Abdominal and pelvic pain | 5 |
|  | Other urinary tract disorders | 5 |
|  | Hypertensive crisis | 5 |
|  | Chronic kidney disease | 4 |
|  | Others | 111 |

**Supplementary Table 3. Costs per patient journey, clinical status and episode during the first year in patients discharged home from the ED**

|  | **Primary HF**  (n= 434) | | | **Secondary HF**  (n= 363) | | | | **P value** |
| --- | --- | --- | --- | --- | --- | --- | --- | --- |
|  | **Frequencies** | **Cost (euros)** | **%** | | **Frequencies** | **Cost (euros)** | **%** |  |
| Total patient-days, n | 158,410 |  | 100% | | 132,495 |  | 100% | - |
| **Total cost** |  | **2,247,217** | 100% | |  | **1,416,579** | 100% | - |
| **Mean cost per patient journey** | | **5,177.9±9,537.3** |  | |  | **3,902.4±6,997.5** |  | <0.001 |
| Mean cost per patient per day | | 14.18±151.82 |  | |  | 10.69±126.49 |  | <0.001 |
| **Use and cost per clinical status** | | | | | | | | |
| Mean days spent at ED | 4.1±3.2 |  |  | | 3.5±2.7 |  |  |  |
| **Emergency Department** |  | **282,410.42** | **12.56%** | |  | **188,482.29** | **13.3%** | - |
| Mean days spent in hospital | 9.2±16.8 |  |  | | 5.8±10.6 |  |  |  |
| **Total costs for hospitalizations** | | **1,888,861.18** | **84%** | |  | **1,105,767.1** | **78%** | - |
| Index hospitalizations |  | 946,899.95 | 42.13% | |  | 658,371.27 | 46.47% | - |
| Readmissions |  | 941,961.23 | 41.9% | |  | 447,395.83 | 31.58% | - |
| **Day-Hospital** |  | **75,945.71** | **3.3%** | |  | **122,329.44** | **8.6%** | - |
| **Mean cost per episode** |  | **1,170.4±3,410.6** |  | |  | **941.9±2,827.6** |  | 0.031 |
| Number of episodes | 1,920 |  | 100% | | 1,504 |  | 100% |  |
| Emergency Department | 1,351 | 209.0±136.0 | 70.3% | | 981 | 192.1±125.1 | 65.2% | 0.003 |
| Day-Hospital | 145 | 523.8±759.6 | 7.5% | | 277 | 441.6±608.6 | 18.4% | 0.008 |
| Hospital | 425 | 4,444.4±6,213.5 | 22.1% | | 246 | 4,495.0±5,775.8 | 16.3% | 0.818 |
| Index hospitalizations | 221 | 4,284.6±5,709.9 | 11.5% | | 148 | 4,448.5±5,060.1 | 9.8% | 0.427 |
| Readmissions | 203 | 4,640.2±6,728.5 | 10.5% | | 98 | 4,565.3±6,741.5 | 6.5% | 0.57 |

**Supplementary Table 4. Top 20 primary diagnoses among hospitalized patients with secondary heart failure diagnosis**

| **Rank** | **Primary diagnosis** | **Episodes**  **n (%)** |
| --- | --- | --- |
|  | Pneumonia, unspecified microorganism | 122 (9.71) |
|  | Other chronic obstructive pulmonary diseases | 101 (8.03) |
|  | Respiratory failure | 81 |
|  | Acute bronchitis | 69 |
|  | Other sepsis | 65 |
|  | Acute lower respiratory tract infection, not elsewhere specified | 54 |
|  | Other respiratory disorders | 42 |
|  | Acute myocardial infarction | 36 |
|  | Atrial fibrillation and flutter | 35 |
|  | Other urinary tract disorders | 32 |
|  | Cerebral infarction | 25 |
|  | Influenza due to other identified types of influenza viruses | 22 |
|  | Iron deficiency anaemia | 19 |
|  | Acute renal failure | 16 |
|  | Pulmonary embolism | 16 |
|  | Pneumonitis due to solids and liquids | 14 |
|  | Other digestive tract diseases | 14 |
|  | Angina pectoris | 13 |
|  | Colelythiasis | 13 |
|  | Non-rheumatic aortic valve disorders | 12 |
|  | Others | 456 |

**Supplementary Figure 1. Flow chart including patient inclusions, exclusions and dispositions according to primary or secondary heart failure diagnoses**

**
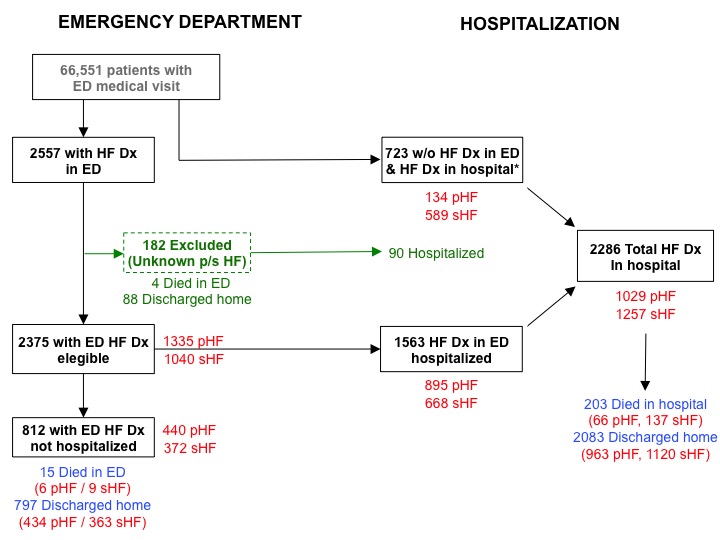
**

* Only when coded as present on admission (POA)

**Abbreviations.** ED, Emergency department; Dx, Diagnosis; HF, Heart failure

pHF, Primary heart failure; sHF, Secondary heart failure

**Supplementary Figure 2. 30-day (top) and 1-year (down) composite outcomes using the COHERENT model according to the type of heart failure diagnosis: primary (left) and secondary (right) in patients hospitalized after the ED**

**
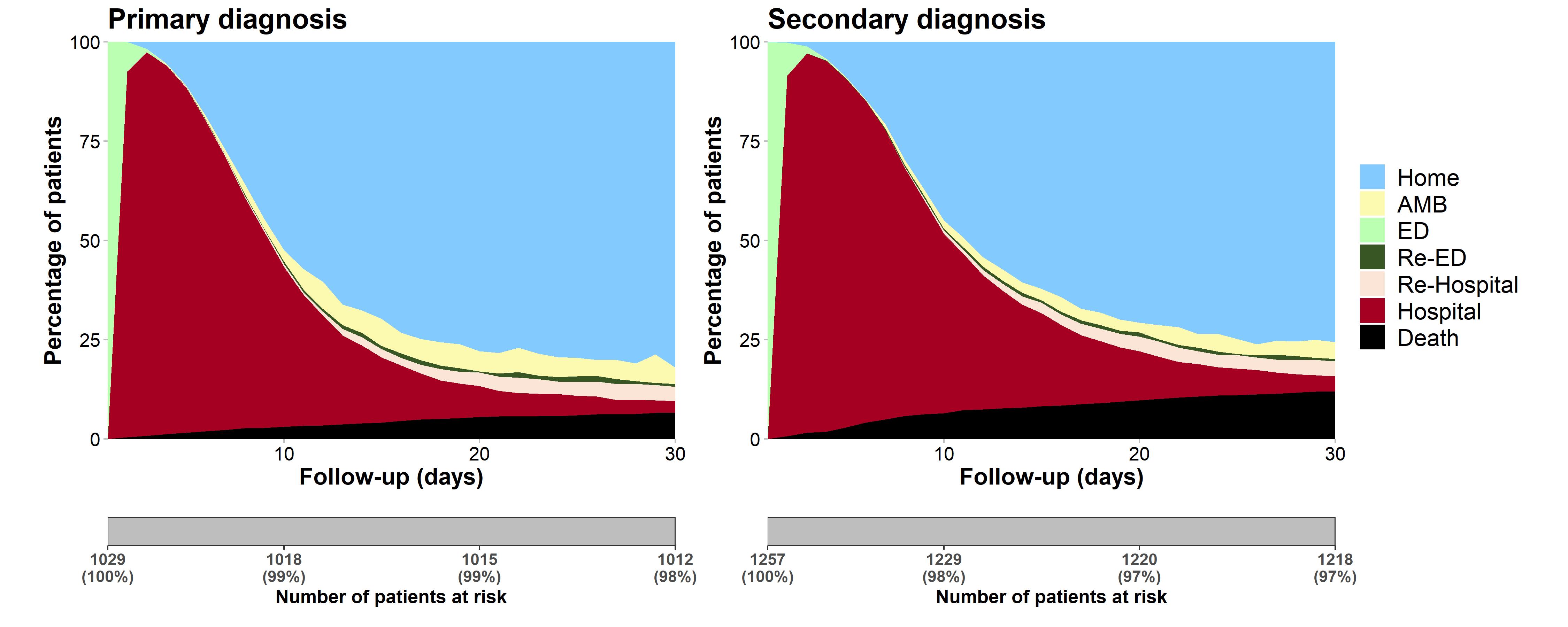
**

**
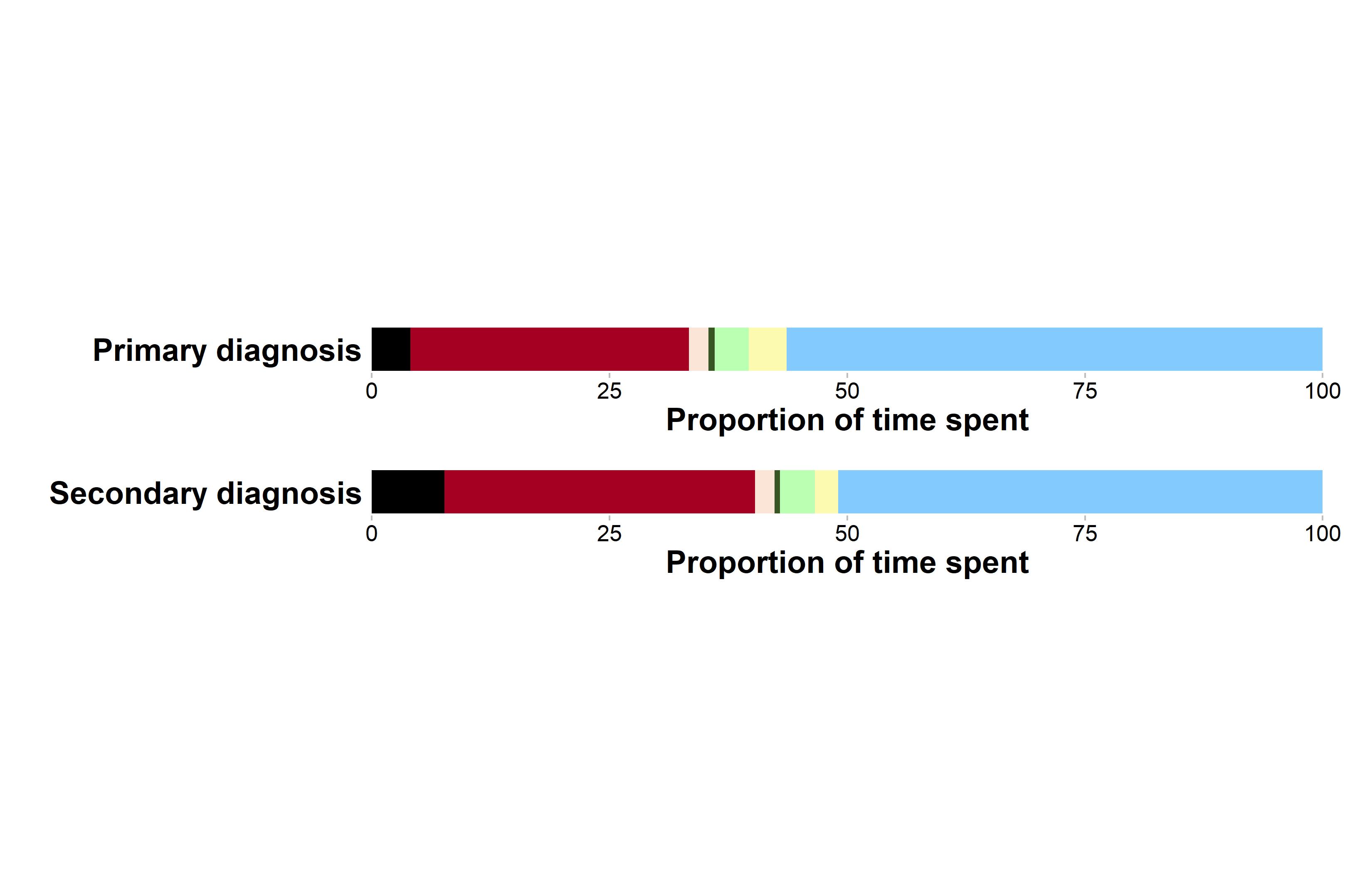
**

**
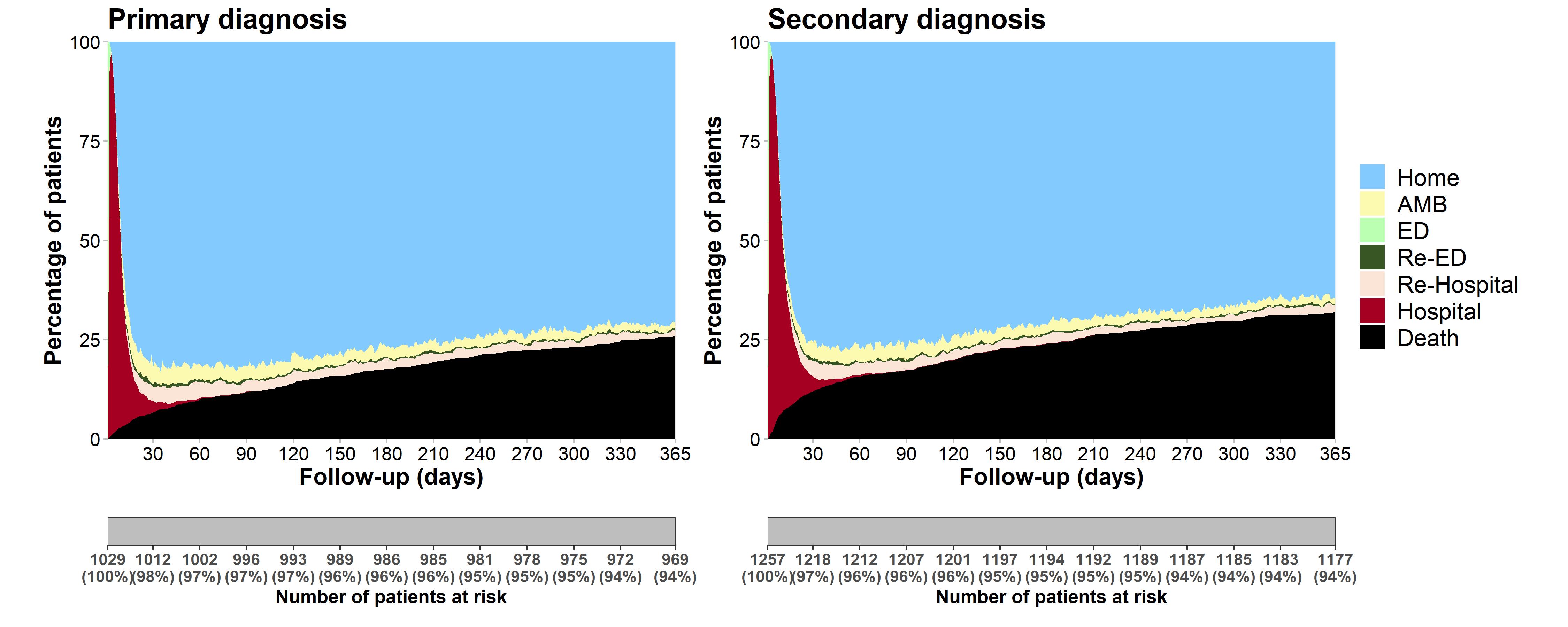

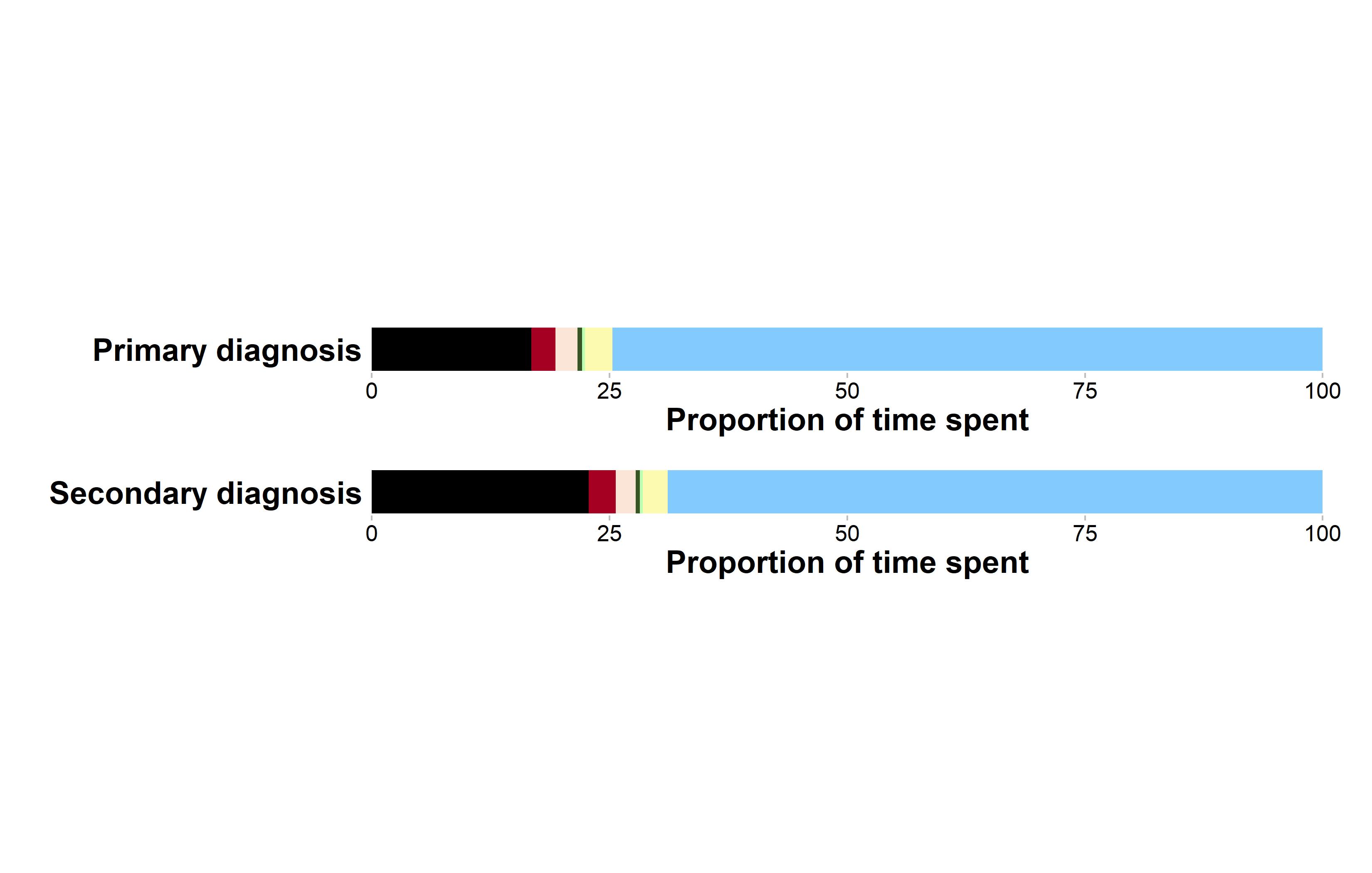
**

**Supplementary Figure 3. 30-day (top) and 1-year (down) composite outcomes using the COHERENT model according to the type of heart failure diagnosis: primary (left) and secondary (right) in patients discharged home from the ED**

**
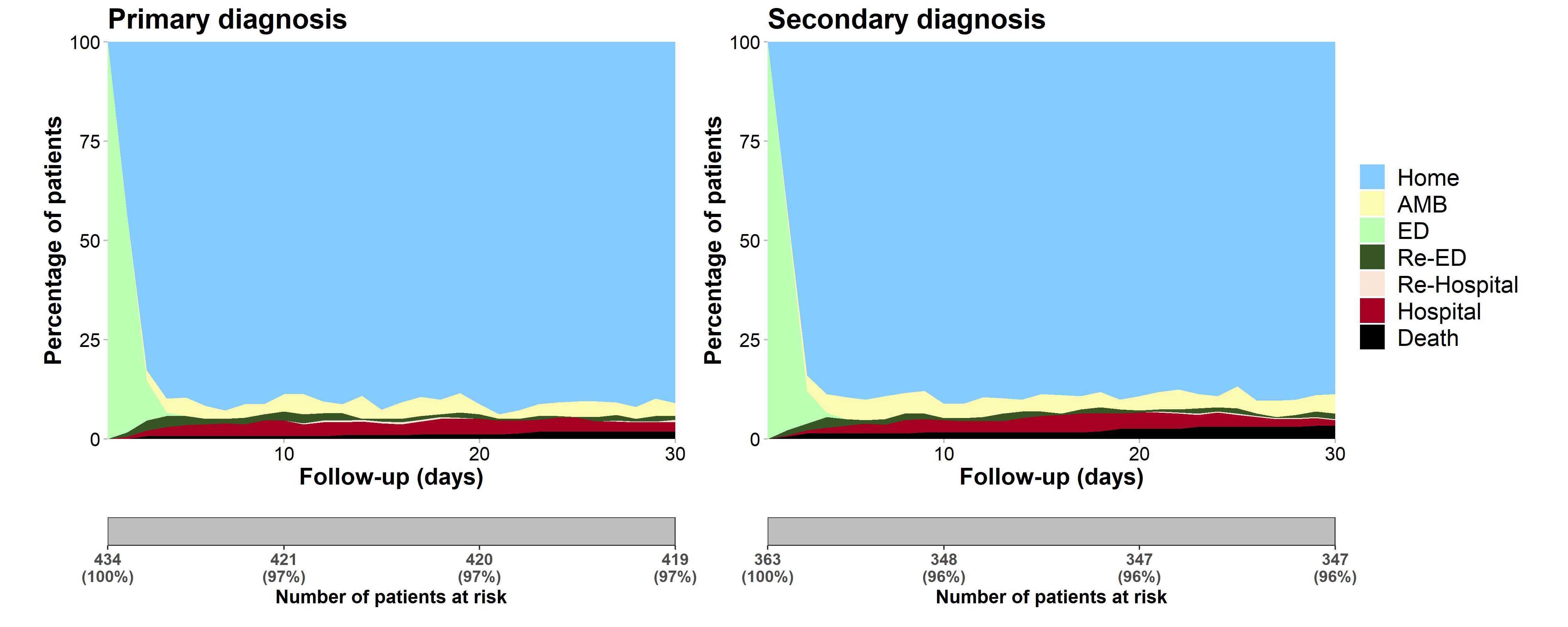
**

**
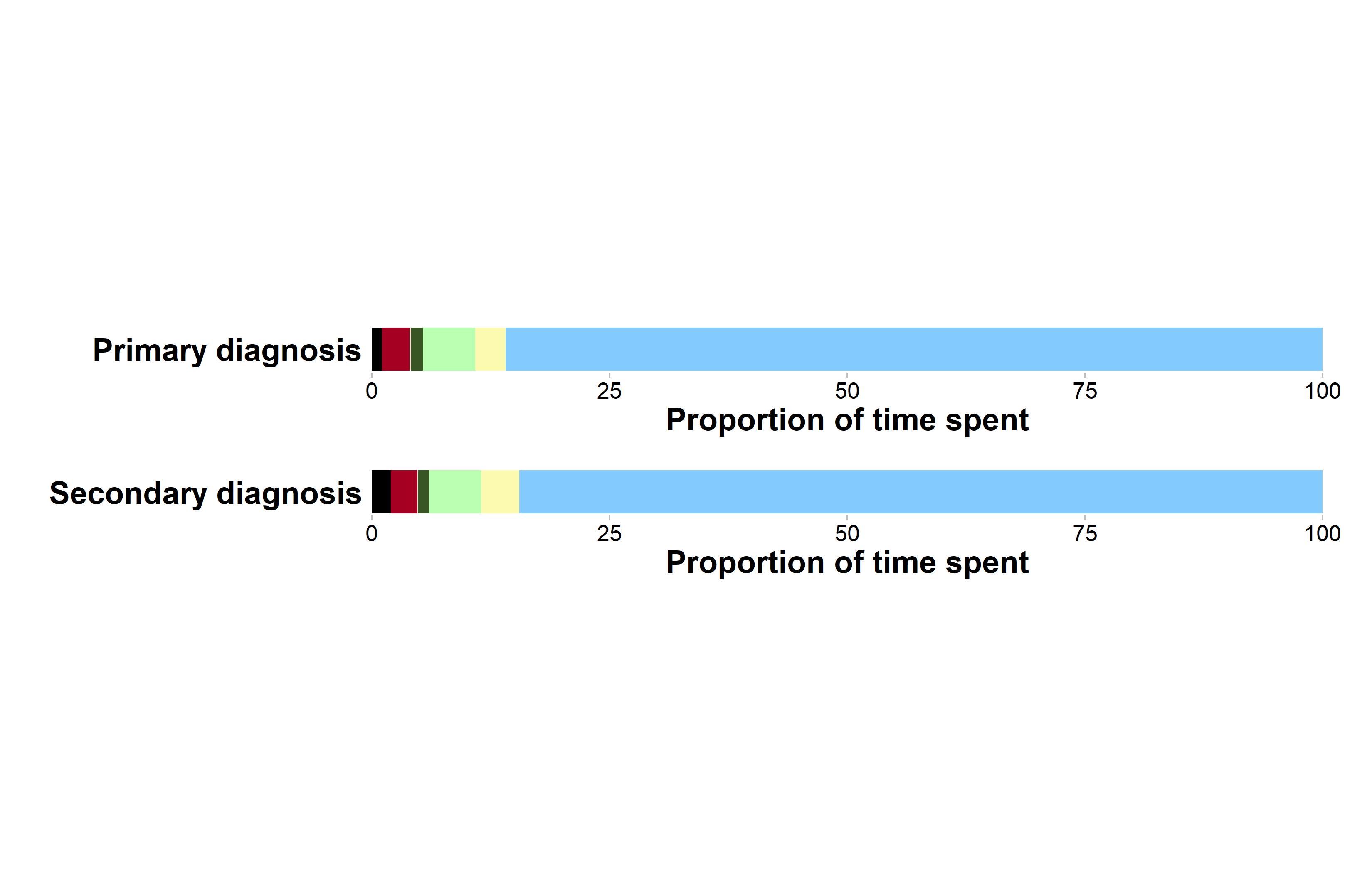
**

**
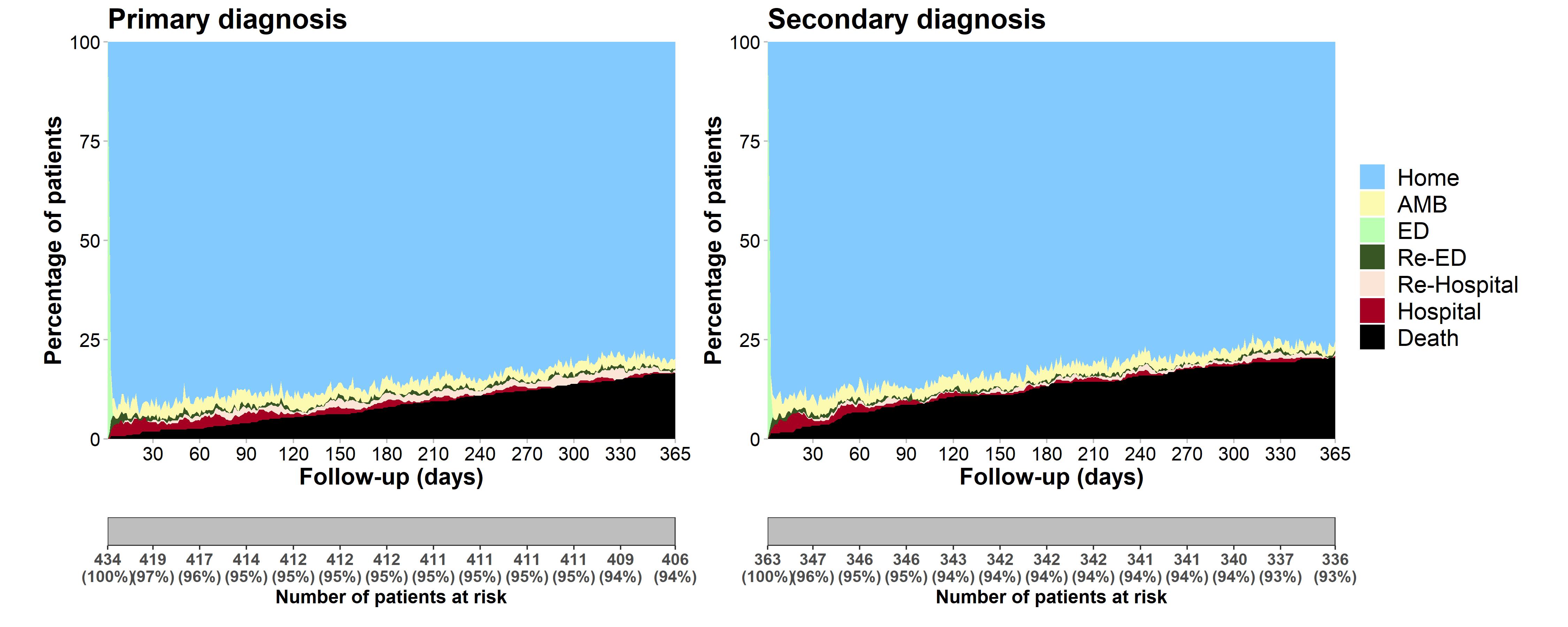
**

**
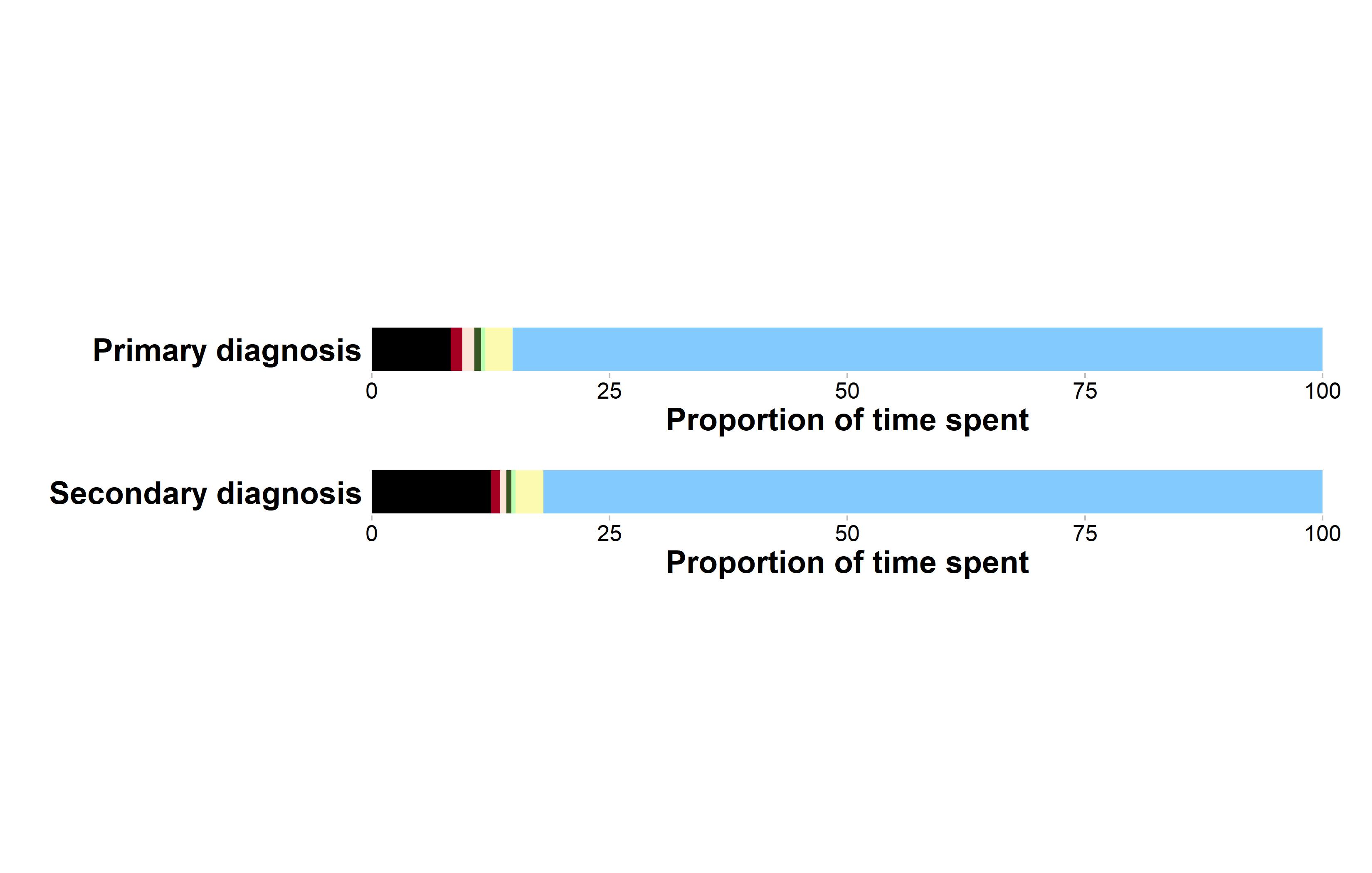
**
